# Bioprotective role of *Cladosporium tenuissimum* counteracting heavy metal (chromium) stress in *Triticum aestivum* L.

**DOI:** 10.3389/fmicb.2025.1685402

**Published:** 2025-11-26

**Authors:** Sabrina Shahid, Ambrin, Shahida Bibi, Sharipova Vasila, Fayaz Asad, Tsanko Gechev

**Affiliations:** 1 Department of Chemical and Life Sciences, Qurtuba University of Science and Information Technology, Peshawar, Pakistan; 2 Center of Plant Sciences and Biodiversity, Women Campus, University of Swat, Charbagh, Pakistan; 3 Institute of Botany, Academy of Sciences, Tashkent, Uzbekistan; 4 Department of Botany, Bacha Khan University, Charsadda, Khyber Pakhtunkhwa, Pakistan; 5 Department Molecular Stress Physiology, Center of Plant Systems Biology and Biotechnology, Plovdiv, Bulgaria; 6 Department of Molecular Biology, Plovdiv University, Plovdiv, Bulgaria

**Keywords:** *Triticum aestivum*, heavy metals, chromium (VI), Cladosporium tenuissimum, plant growth, phyto-hormones, antioxidant enzymes

## Abstract

Heavy metal pollution, particularly from chromium (Cr), poses a significant threat to plant development and soil health. This study evaluated the efficacy of the rhizospheric fungus strain *Cladosporium tenuissimum*, isolated from the rhizosphere of *Cannabis sativa*, in enhancing the early growth and physiological resilience of *Triticum aestivum* L. (wheat) under chromium-induced stress. The strain *Cladosporium tenuissimum* demonstrated tolerance through substantial biomass retention in Czapek-Dox medium at chromium concentrations between 30 and 90 mg/L, mitigated the decrease in carotenoid and chlorophyll pigments, enhanced protein and sugar levels, and significantly promoted root and shoot growth in wheat under chromium stress. Additionally, fungal therapy reduced proline accumulation and restored indole-3-acetic acid (IAA) levels, indicating improved hormonal balance and reduced stress levels. Furthermore, *Cladosporium tenuissimum* (*C. tenuissimum*) maintained membrane integrity and regulated excess flavonoids by reducing electrolyte leakage. Under chromium stress, the activities of antioxidant enzymes were elevated; however, fungal inoculation moderated these activities to some extent. These findings indicate that *C. tenuissimum* may serve as a bioprotective agent, enhancing plant tolerance and mitigating Cr toxicity by regulating metabolic processes, physiological responses, and antioxidant activity.

## 1 Introduction

Large-scale industrialization has contributed significantly to the increase in environmental pollution in recent decades by introducing heavy metals (HMs) into the soil, water, and atmosphere (Ertani et al., 2017; Younas et al., 2022). Electroplating, tanning, textile dyeing, metallurgy, and mining are significant anthropogenic sources of heavy metal (HM) contamination (Bello et al., 2023; Jagaba et al., 2024). Chromium (Cr) is a highly hazardous and persistent contaminant. Hexavalent chromium [Cr(VI)] poses significant concerns because of its increased mobility and toxicity to plants, being 100 times more detrimental than its trivalent counterpart (Sharma et al., 2022). In developing nations such as Pakistan, Bangladesh, and certain regions of Africa, untreated wastewater from industries containing hexavalent chromium [Cr(VI)] is frequently utilized for irrigation, particularly in periurban agricultural zones (Vilardi et al., 2018; Rahman and Singh, 2019; Chen et al., 2022). Chromium contamination in soils is concerning because of its presence in two oxidative states: Cr(III) (Apte et al., 2006), which is relatively less harmful and less mobile, and Cr(VI), which is highly mobile, bioavailable, and toxic to plants (Laxmi and Kaushik, 2020). The uptake of Cr(VI) into plant roots, leading to its rapid translocation to aerial tissues, where it interferes with various biological and physiological processes (Zulfiqar et al., 2023). Excessive exposure to chromium disrupts cellular redox balance, induces lipid peroxidation, damages chloroplasts, impairs photosynthesis, and adversely affects proteins (Hayat et al., 2012). It also adversely affects the germination, tillering, biomass accumulation, grain yield, and food safety of *Triticum aestivum* L. (wheat) (Arshad et al., 2017), a globally significant crop and staple food. Because of the persistence of Cr(VI) in the environment and its ability to migrate through soil and groundwater over significant distances, it is crucial to identify effective, durable, and cost-efficient remediation methods.

Biological remediation, particularly microbe-assisted phytoremediation, has gained popularity because of its cost-effectiveness, environmental benefits, and scalability. Rhizospheric fungi are increasingly recognized as effective bioremediators of soils contaminated with heavy metals (Mishra et al., 2017; Refaey et al., 2021). This is because of their extensive hyphal networks and ability to adapt their metabolism to varying conditions. Fungi mitigate metal toxicity through biosorption, redox transformation, chelation with organic acids and siderophores, and secretion of exopolysaccharides that capture toxic ions (Arwidsson et al., 2010; Saharan et al., 2023). Fungal inoculants facilitate metal detoxification in plants while enhancing growth through improved nutrient uptake, modulation of phytohormonal balance, and augmentation of plant antioxidant defenses (Liu et al., 2022). It may also have the ability to withstand elevated concentrations of Cr(VI), convert it into the less toxic Cr(III) form in soil and water, and enhance plant growth under stress by altering photosynthesis, osmolyte production, and enzyme utilization (Gil-Cardeza et al., 2018; Rabab et al., 2025).

Despite these promising results, there is limited research on the application of the rhizofungus *Cladosporium tenuissimum* to mitigate chromium toxicity in wheat. Previous studies have primarily focused on bacterial inoculants and model plants. Limited research has examined the interactions between filamentous fungi and major grain crops under heavy-metal stress. Investigating the physiological, biochemical, and oxidative stress responses of wheat to fungal-assisted chromium remediation is crucial, particularly in regions where contaminated wastewater is frequently utilized for irrigation. This study determined the tolerance of a rhizospheric isolate of *C. tenuissimum* to Cr(VI) and assessed its ability to reduce Cr uptake in *Triticum aestivum* L. and mitigate toxicity under graded Cr stress, evaluating its impact on key physiological traits, antioxidant enzyme activity, and biomass production.

## 2 Materials and methods

### 2.1 Isolation and culturing of rhizosphere fungi

*Cannabis sativa* plants were collected from specified locations (roadside) in Charsadda District, KP, Pakistan, by excavating them with a spade, including the adjacent soil. The roots were vigorously agitated in the air to eliminate excess soil. Rhizosphere soil was collected by agitating the roots in a 1 L beaker containing autoclaved distilled water. A serial dilution method was employed to isolate soil-borne fungi in the rhizosphere (Soylu et al., 2005), with dilutions ranging from 10^−2^ to 10^−5^. To inhibit bacterial proliferation, 20 mL of potato dextrose agar (PDA) containing streptomycin (2.50 mg/mL) was aseptically dispensed into sterile Petri dishes. Each dilution was subjected to the application of a 100 μL aliquot, which was uniformly distributed across the agar surface utilizing a spreader. Following sealing of the plates with Scotch tape, they were incubated at 28 °C for a duration of 3 to 7 to 10 days. The fungal colonies were meticulously extracted from their petri dishes and subsequently transferred to PDA plates and incubated for 7 days at 28 °C ± 2 °C. The pure PDA culture slants were stored at 4 °C for subsequent use.

### 2.2 Evaluation of fungal isolates for Cr(VI) tolerance

The isolates were grown in 500 mL flasks with 50 mL of Czapek’s (CZ) medium in order to evaluate their resistance to Cr (VI) (hereafter referred to as Cr). The Czapek medium contained potassium dichromate (Cr) at concentrations of 100, 300, 600, and 1,200 μg as the source of Cr. Three replicates were used for each concentration, in addition to metal-free controls. The radial growth of the fungal isolates was evaluated at varying concentrations of heavy metals. The cultures were maintained in a shaking incubator at 28 °C and 120 rpm for 7–10 days. Following the incubation period, filtration was performed to separate the biomass from the culture filtrate. Biomass exhibiting the greatest tolerance to heavy metals was chosen for the final experiment.

### 2.3 Molecular identification of the rhizospheric fungal isolates

DNA extraction from frozen mycelia of a rhizospheric fungal isolate was conducted following the method described by Chi et al. (2009) for molecular identification. PCR was used to make copies of the ITS 1 and 4 parts of the DNA. There were 10 μL of PCR master mix, 1 μL of DNA sample, 1 μL of each primer, and 7 μL of double-distilled water in the reaction mixture, which had a total volume of 20 μL. The PCR process started with a 2-min denaturation at 95 °C, followed by a 1-min annealing at 55 °C, and then a 5-min extension at 72 °C. This was done 35 times. Then, a sequencing laboratory sequenced the amplified fragment. We used NCBI BLAST^1^ to compare the final consensus sequence with nearly comparable sequences in GenBank. MEGA-11 was used to assess the phylogenetic relationship of the ITS sequence of our isolate by examining the close hits (Keklik, 2023).

### 2.4 Experimental design and treatments

The experiments were conducted in a greenhouse at Qurtuba University in Peshawar, utilizing natural light. The temperature ranged from 22 °C to 28 °C, and the humidity varied between 60.0 and 65.55%. The light cycle lasted for approximately 12–13 h. Prior to the experiment, soil samples were collected from a farm area adjacent to a road in Serdhari. The soil composition consisted of sandy loam, exhibiting a pH of 7.78 and an electrical conductivity of 266 μS cm^−1^. Nutrient analysis revealed 94.75 mg kg^−1^ of nitrogen, 9.50 mg kg^−1^ of phosphorus, and 110.34 mg kg^−1^ of potassium. The extractable chromium (Cr) using DTPA was quantified at 2.31 mg kg^−1^, existing as potassium dichromate (K₂Cr₂O₇).

A stock solution of potassium dichromate was prepared using double-distilled water to achieve a concentration of 10.0 g/L. The surface of *Triticum aestivum* L. seeds was sterilized, followed by planting in autoclaved plastic pots at a seed-to-pot ratio of 3:1. The experiment consisted of three replicates, and the design was entirely random. The treatments included the addition of potassium dichromate to the soil at concentrations of 100–200 μg/mL, application of a rhizospheric fungal strain, and utilization of the fungus with diminished Cr levels (30, 60, and 90 μg/mL). Distilled water was applied to the pots as necessary to maintain a consistent soil moisture level throughout the trial. The quantities of Cr were selected based on the initial screening tests. Cr was incorporated into the soil prior to seed sowing. *Triticum aestivum* L. seedlings were regularly irrigated and harvested at three-leaf stages, approximately 15 days after sowing, for assessing various growth and biochemical analyses.

### 2.5 Morphological and physiological analysis

#### 2.5.1 Morphometric exploration

The wheat seedlings were gently removed from their pots, and blotting paper was used to dry them off after washing in a pail of water to remove any remaining soil. After 15 days (three-leaf stage), we used a measuring scale to determine the root and shoot lengths of the plants and a digital balance to obtain the fresh weight. To determine the dry weight, the samples were dried in an oven at 70 °C until they reached a consistent weight.

#### 2.5.2 Determination of photosynthetic, sugar, and protein contents

To estimate photosynthetic pigments, fresh wheat leaves were extracted in 80.0% acetone, and the extract was assessed for absorbance at 480, 645, 663, and 510 nm using a UV–Vis spectrophotometer. Chlorophyll quantities were quantified using the method described by Arnon (1949) as follows:


Chla(μmg/mL−1)=(12.25A663−2.79A647)

Chlb(μmg/mL−1)=(21.50A647−5.14A663)

TotalChl(μmg/mL−1)=Chla+Chlb

$$\begin{array}{l} \text{Carotenoids}\;(\mathrm{\mu gm}L^{-1})\;\\ =\frac{(1000\;A470-1.82\;\text{Chlorophyll}\;a-85.02\;\text{Chlorophyll}\;b)\;\;}{198} \end{array}$$

The protein concentration of wheat leaves was determined using the Bradford technique (Bradford, 1976), whereas total sugar was observed using the method described by Gao et al. (2013). Absorbance was measured at 620 nm using a spectrophotometer.

#### 2.5.3 Determination of IAA, proline, and flavonoids concentrations

The Salkowski solution was used to measure IAA in wheat leaves; 50.0 mL of perchloric acid and 1 mL of ferric chloride were mixed to prepare the Salkowski reagent. To study plant matter, a pestle and mill were used to break fresh wheat (0.5 g) with 3 mL of ethanol. A speed of 10,000 rpm was used to spin the solution for 15 min. There was 1 mL of residue and 2 mL of Salkowski reagent in a test tube. The tube was kept at room temperature for 30 min. The optical density was measured at 540 nm (Hussain et al., 2017).

The proline content in fresh wheat leaves was assessed using the method established by Bates et al. (1973). Fresh leaf tissue (0.2 g) was homogenized in 5 mL of 3% sulfosalicylic acid and stored at 5 °C for 24 h. The extract was centrifuged at 4000 rpm for 5 min. Two milliliters of the supernatant was combined with 2 mL of acid ninhydrin reagent in individual 10 mL test tubes. Ninhydrin was dissolved in 20 mL of glacial acetic acid and 20 mL of 6 M phosphoric acid to prepare the acid ninhydrin. The mixture was incubated in a water bath at 100 °C for 60 min and then cooled to room temperature. Subsequently, 4 mL of toluene was introduced into each sample, and the toluene layer containing the chromophore was isolated. Absorbance was recorded at 520 nm, with toluene serving as the blank and a reference.

Colorimetric analysis was used to assess the flavonoid content of the plants. A quantity of 0.5 mL of methanol was employed (Chang et al., 2002). The mixture was vortexed and incubated for 30 min at room temperature. It contained 0.5 mL of leaf extract, 100 μL of potassium acetate (1 M), 100 μL of aluminum chloride (10%), and 4.8 mL of methanol (80%). The assessment of optical density was conducted at 415 nm, using methanol as a blank.

### 2.6 Biochemical analysis

#### 2.6.1 Analysis of antioxidant enzyme activity

The extraction and determination of antioxidant enzymes from the seedlings were conducted as previously described (Alici and Arabaci, 2016). Enzyme extraction and quantification were performed as previously described. Frozen seedling leaves (1 g) were extracted using phosphate buffer (pH 7). The supernatant was obtained by centrifugation at 12,000 × *g* for 15 min at 4 °C. The riboflavin/nitroblue tetrazolium (NBT) method was employed to estimate superoxide dismutase (SOD) activity and measure the resultant product at 560 nm. The peroxidase (POD) activity was assessed using the o-dianisidine method, with the optical density of the product measured at 470 nm. The activity of catalase (CAT) (Anderson, 2002) was assessed using H_2_O_2_ as a substrate. Standard curves were used for the quantification of enzyme activities, which were expressed as micrograms per milligram of protein.

#### 2.6.2 Assessment of catalase activity and electrolyte leakage

Catalase (CAT) content in wheat leaves was determined using Lück’s (1965) methodology. Fresh wheat leaves, weighing 0.2 g, were triturated with 10 mL of phosphate buffer utilizing a pestle and mortar. The solution was then centrifuged for 5 min at 10,000 rpm. Three milliliters of hydrogen peroxide-phosphate buffer were mixed with 0.4 milliliters of leaf extract supernatant. Optical density was measured at 240 nm. Hydrogen peroxide was used as a blank (Wheeler et al., 1990).

The wheat leaf sample underwent three washes with distilled water. After dividing the leaf into five uniform circular discs, the discs were placed in test tubes with 10 mL of distilled water and agitated at 5-min intervals. Following a 24-h incubation period at room temperature in the absence of light, the initial electrical conductivity (EC1) was recorded using an EC meter. Following a 15-min exposure to 120.0 °C in an autoclave, the discs were prepared for a second electrical conductivity (EC2). The following formula was employed to determine the final result, expressed as a percentage:


$$\mathrm{EC}=\frac{\mathrm{EC}1}{\mathrm{EC}2}\times 100$$

### 2.7 Statistical analysis

The replicated data were analyzed through a one-way analysis of variance (ANOVA) utilizing the Statistical Package for the Social Sciences (SPSS) 16.0 software. In cases where significant differences were identified, pairwise comparisons were conducted using Duncan’s multiple range test. A Venn diagram was created to illustrate the metabolites that were differentially expressed across the control, Cr-stressed, and Cr + *C. tenuissimum* treatments, highlighting both similarities and differences. Differential features were selected using ANOVA (*p* < 0.05) and a fold-change threshold of ≥2. The VennDiagram package in R (version 4.3.0) was used to create diagrams.

## 3 Results

### 3.1 Growth of the isolated fungus strain and its characterization

In order to isolate the rhizospheric fungus, *Cannabis sativa* was used as the host plant. Pure colonies were obtained by successfully recovering and cultivating five different fungal strains, which were identified as dark grayish (SGRF), green (SGRF), white (SWRF), black (SBRF), and light bluish (SBRF; S stands for Sabrina, R for rhizosphere, and F for fungus) on PDA medium. Each isolate was then cultivated on Czapek-Dox medium supplemented with different concentrations of Cr for preliminary screening in order to evaluate their resistance to heavy metal stress. The SGRF isolate, which was subsequently determined to be *C. tenuissimum*, showed the greatest resistance to Cr. The biomass production (g/L) of *C. tenuissimum* (CT) at various Cr concentrations: 60.13 g/L at Cr-30 + CT, 54.42 g/L at Cr-60 + CT, 50.69 m/L at Cr-90 + CT, and 57.6 m/L in control (CT alone).

**Figure 1:**
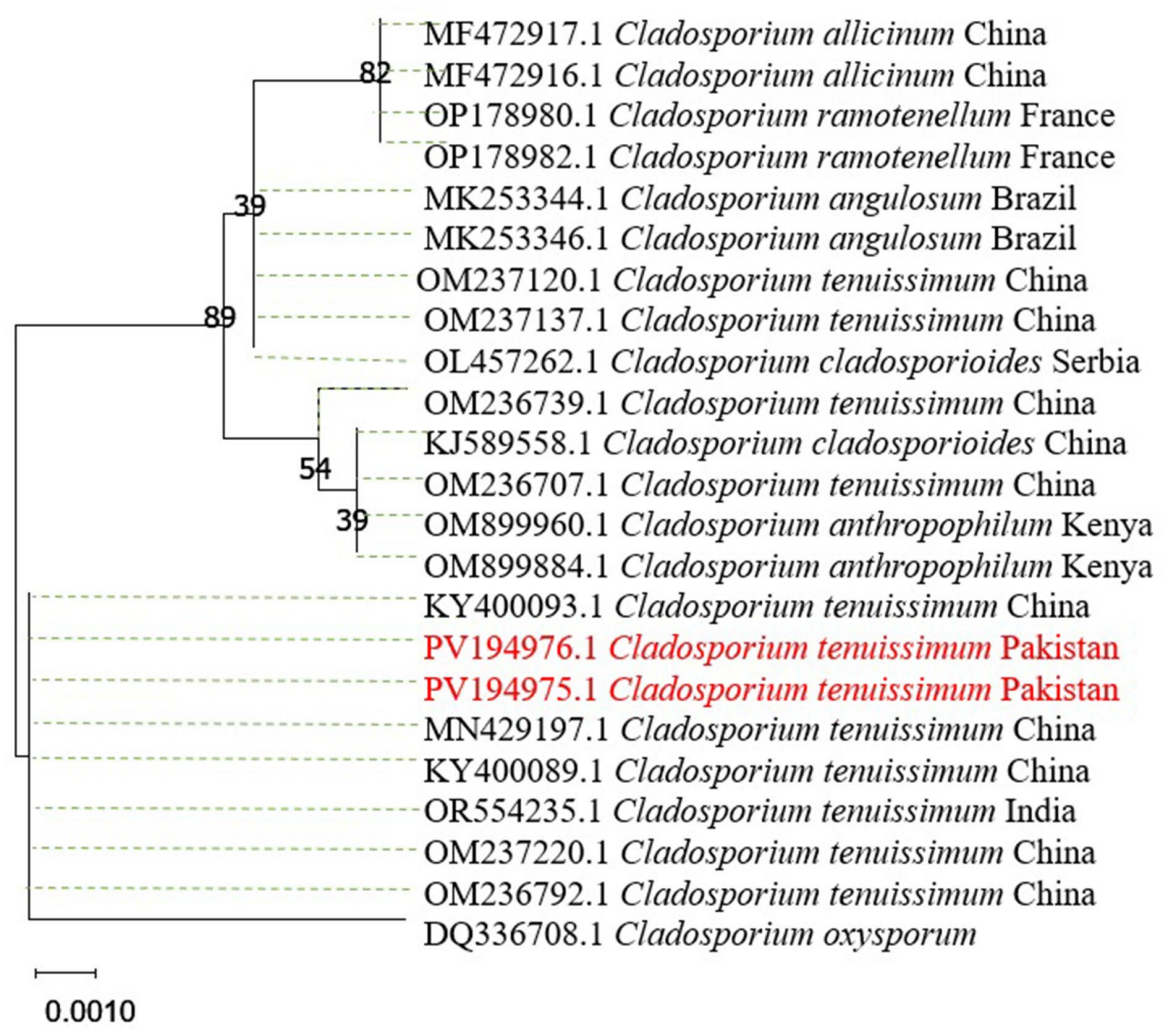
The phylogenetic tree for *C. tenuissimum* was inferred using the maximum likelihood technique (Kimura 2 + with gamma distributions (+G), 10,000 bootstrap).

Morphological characterization was performed on the rhizospheric fungal isolate *C. tenuissimum*, which was chosen for its capacity to promote plant development in wheat seedlings and its resistance to chromium. On PDA medium, this isolate produced characteristic olivaceous-brown to blackish-brown colonies. As the culture grew older, the colony color progressively changed from white to dark brown, a trait common to the *Cladosporium* genus. The physical and molecular characteristics supported *C. tenuissimum* genus-level identification by matching the descriptions of the species (Figure 1).

The maximum likelihood inferred phylogenetic tree of *C. tenuissimum* produced several clades of the genus *Cladosporium* spp. The haplotype in the current study was grouped with similar species reported in China (MN429197 and KY400089). The *C. tenuissimum* sequence also clustered with *C. tenuissimum* reported from India (OR554235). The tree used several other species of the same genus to reference the current haplotype. All *C. tenuissimum* were clustered with 99.8% ultra-fast bootstrap support values and a 97% approximate likelihood ratio, iterated 1,000 times. *C. oxysporum* species (DQ336708) was used as an outgroup for reference, as shown in Figure 1.

### 3.2 Morphological and physiological responses

#### 3.2.1 Morphological responses of wheat seedlings

The growth performance of wheat was evaluated under varying Cr stress levels and fungal inoculation with *C. tenuissimum* by measuring root number, root length, shoot length, and leaf count. Leaf number was consistently recorded at two across all treatments, demonstrating no significant impact of chromium or fungal treatment on leaf initiation during the initial growth phase. A dose-dependent reduction in root number, root length, and shoot length was evident under chromium stress (Cr-30, Cr-60, and Cr-90) compared to the untreated control. The root number decreased from 2.67 ± 0.58 in the control group to 2 ± 0, 2.03 ± 0.58, and 1.67 ± 1.15 in the Cr-30, Cr-60, and Cr-90 treatments, respectively. Root length decreased from 3.43 ± 1.50 cm (control) to 2.40 ± 0.44 cm, 2.13 ± 0.59 cm, and 1.86 ± 0.92 cm with increasing Cr levels. Shoot length exhibited a decreasing trend, declining from 2.06 ± 0.31 cm in the control group to 1.70 ± 1.31 cm (Cr-30), 1.89 ± 0.85 cm (Cr-60), and 1.57 ± 1.16 cm (Cr-90). These results indicate that chromium toxicity negatively impacts root and shoot development in a concentration-dependent manner. Conversely, plants treated solely with *C. tenuissimum* exhibited notable enhancement in all assessed parameters. The root number increased to 4 ± 1, root length to 3.73 ± 0.21 cm, and shoot length to 2.33 ± 0.75 cm, surpassing the control group and indicating a significant growth-promoting effect of the fungal inoculant.

The co-application of *C. tenuissimum* under chromium stress not only alleviated the toxic effects of chromium but also promoted growth relative to chromium treatments alone. In Cr-30 + *C. tenuissimum*, the root number increased to 3.67 ± 1.15, while root and shoot lengths improved to 3.47 ± 0.95 cm and 2.37 ± 0.38 cm, respectively. The Cr-60 + *C. tenuissimum* treatment exhibited the most significant response, achieving the highest root length (4.40 ± 1.01 cm) and a considerable improvement in shoot length (2.27 ± 0.68 cm) relative to Cr-60 alone. At the highest chromium level (Cr-90), the presence of *C. tenuissimum* resulted in an average root number of 3.83 ± 0.58, with root and shoot lengths measuring 3.46 ± 1.26 cm and 2.10 ± 0.80 cm, respectively.

#### 3.2.2 Effect of Cr and *Cladosporium tenuissimum* on photosynthetic pigments

**Figure 2:**
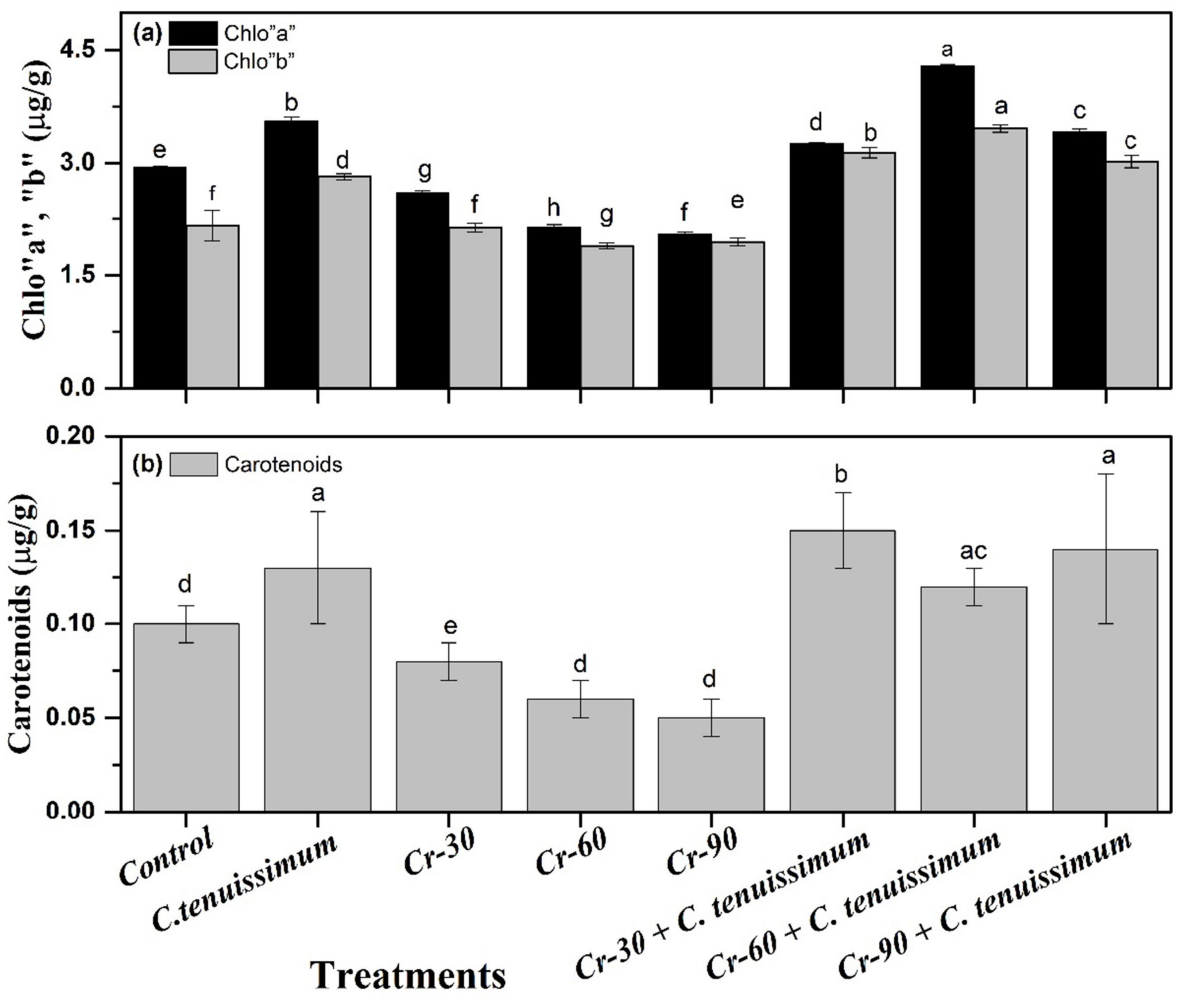
Effect of Cr and *C. tenuissimum* on Chl-a and b **(a)** and carotenoid content **(b)** in wheat. Data are from three biological replicates, mean values ±SD.

Figure 2 presents the impact of chromium (Cr) stress and *C. tenuissimum* application on chlorophyll a (Chl. a) and chlorophyll b (Chl. b), and carotenoids in wheat leaves. Panel (a) shows a significant decline in both Chl. a and Chl. b levels under chromium stress. The Cr-60 treatment exhibited the lowest concentrations, with Chl. a decreasing to approximately 30.64% and Chl. b decreasing to below 17.03%. The Cr-60 + *C. tenuissimum* treatment exhibited the highest pigment accumulation, with Chl. a reaching approximately 45.58% and Chl. b exceeding 60.19%. Fungal inoculation alone, in the absence of chromium stress, also increased chlorophyll levels relative to the control, albeit to a lesser extent than in the combined treatment groups. The combined treatments (Cr-30, Cr-60, and Cr-90 + *C. tenuissimum*) consistently elevated the pigment content compared to the respective Cr-only treatments, suggesting a protective function of the fungus in mitigating Cr-induced pigment degradation (Figure 2a).

Carotenoid content across various treatments. Cr stress alone considerably reduced carotenoid levels, with the lowest concentrations being recorded in the Cr-60 and Cr-90 treatment groups. The application of *C. tenuissimum* notably increased carotenoid content, particularly in the Cr-30 + *C. tenuissimum* treatment, which exhibited the highest carotenoid accumulation (~0.15 μg/g; 50.12%) (Figure 2b). Cr-60 and Cr-60 with a combination of *C. tenuissimum* treatments closely followed, indicating a dose-responsive increase in pigment biosynthesis under fungal protection.

#### 3.2.3 Protein and sugar contents

**Figure 3:**
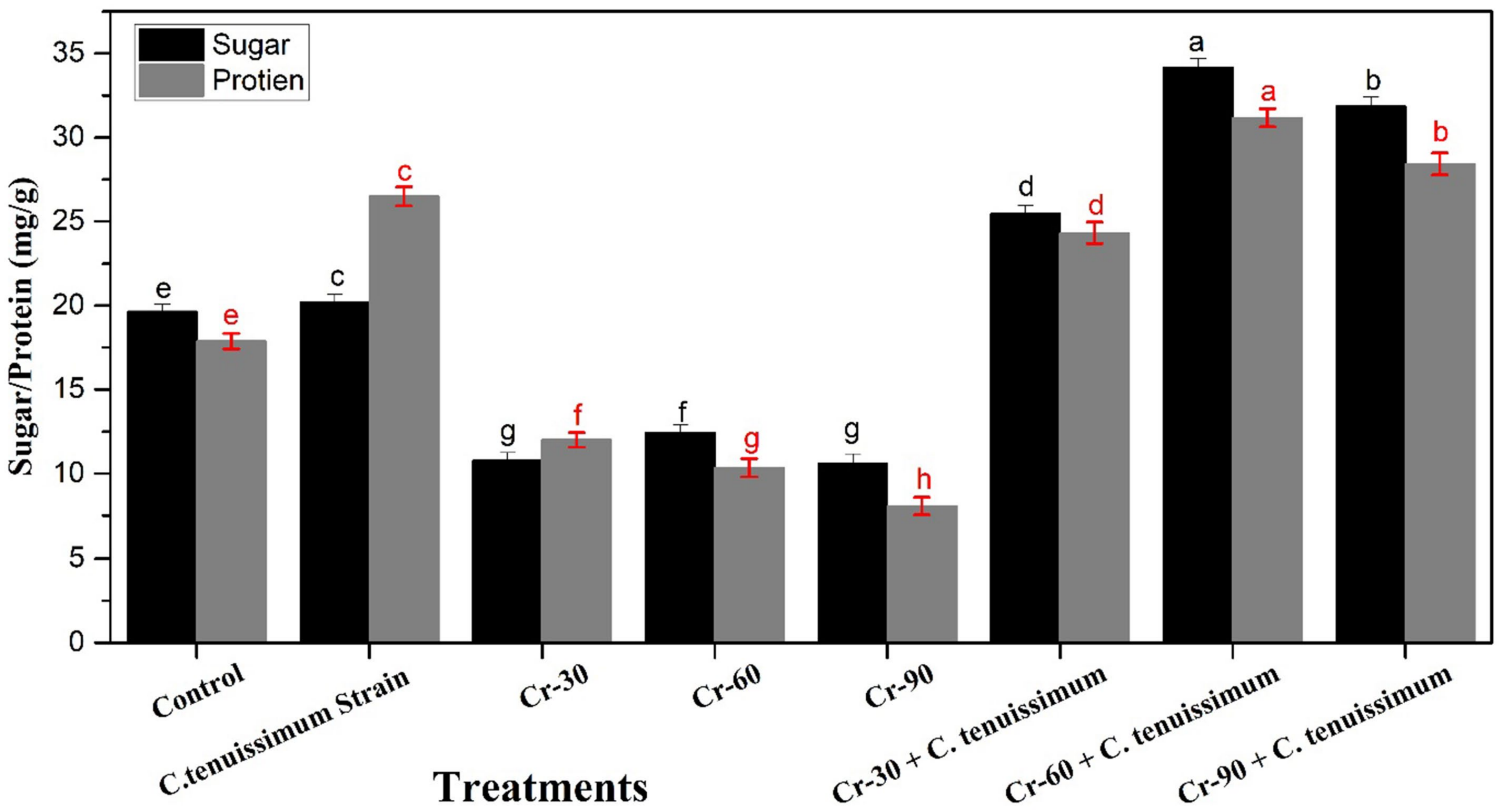
Effect of Cr and *C. tenuissimum* on protein and sugar accumulation in wheat. Data are from three biological replicates, mean values ±SD.

The effects of Cr and *C. tenuissimum* inoculation on sugar and protein content in wheat leaves are shown in Figure 3. Cr stress substantially decreased both sugar and protein levels, whereas the application of *C. tenuissimum,* particularly in conjunction with Cr treatment, markedly enhanced these biochemical parameters. The sugar and protein contents in the control groups were measured at 19.64 mg/g and 17.88 mg/g, respectively. The application of *C. tenuissimum* resulted in a modest increase, with protein content up to 48.21% (26.50 mg/g) and sugar content rising to 2.85% (20.02 mg/g), indicating its inherent growth-promoting potential. The application of Cr stress, specifically at Cr-60 and Cr-90 concentrations, resulted in a reduction in both parameters. Cr-90-treated plants exhibited the lowest values, with sugar content decreasing to 45.93% (10.62 mg/g) and protein content to 54.87% (8.07 mg/g).

The application of *C. tenuissimum* with Cr resulted in a significant increase in sugar and protein levels. In Cr-30 + *C. tenuissimum*, the sugar and protein contents increased relative to Cr-30 alone, achieving values of 29.74% (25.48 mg/g) and 36.07% (24.33 mg/g), respectively. Cr-60 and Cr-90 with a combination of *C. tenuissimum* treatments exhibited the highest values compared to individual treatments of Cr, revealing the significant bioprotective and metabolic enhancement capabilities of *C. tenuissimum* in response to moderate Cr stress (Figure 3).

#### 3.2.4 Indole-3-acetic acid (IAA) and proline concentrations

**Figure 4:**
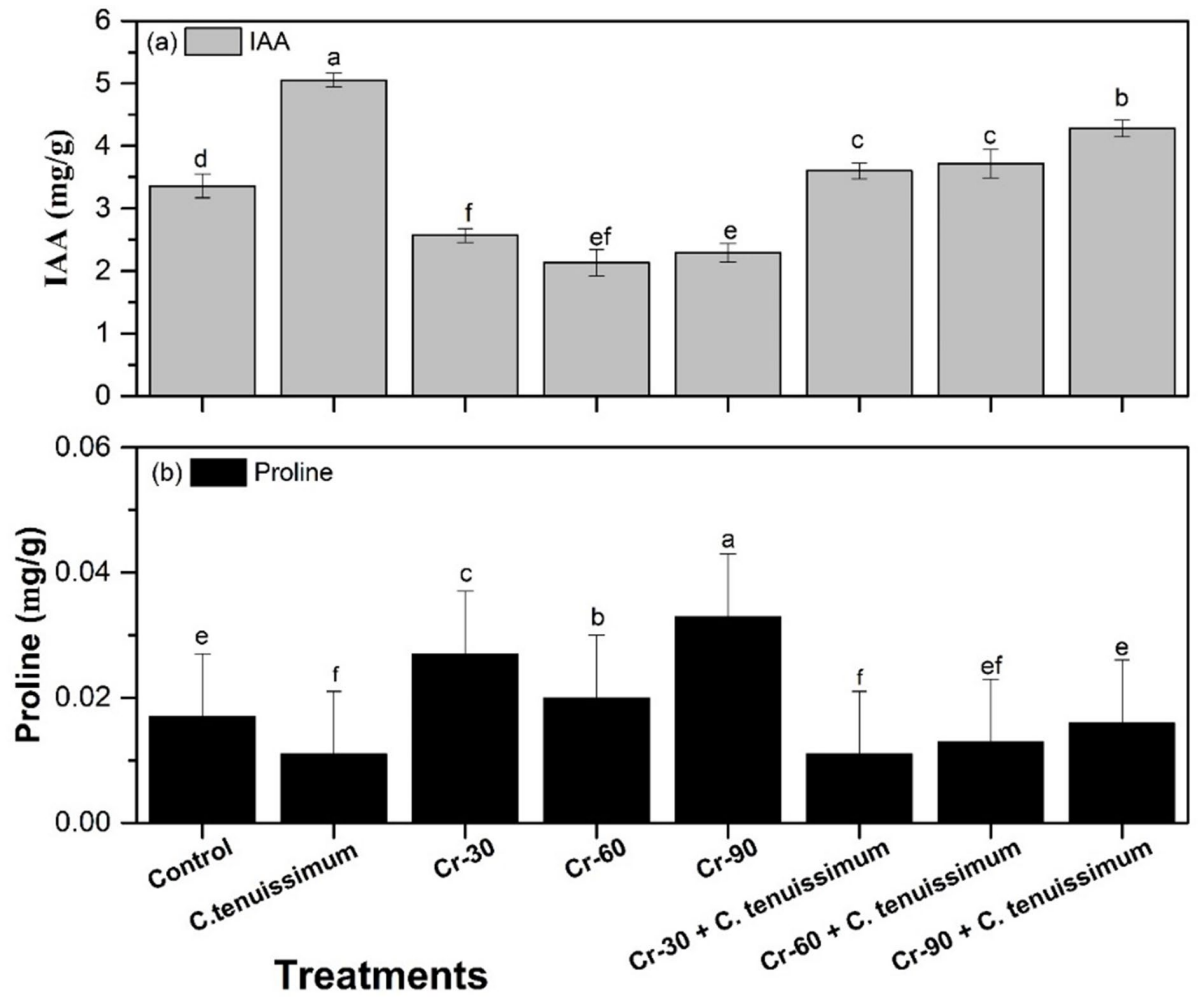
Effect of Cr and *C. tenuissimum* on IAA and protein content of wheat. Data are from three biological replicates, mean values ±SD.

IAA levels varied significantly across treatments (Figure 4a). The highest IAA accumulation was observed in plants treated with *C. tenuissimum*, reaching 50.30% (5.05 mg/g), which was significantly higher than all other treatments (*p* < 0.05). These findings highlight the auxin-producing potential of this fungal strain under normal physiological conditions. In contrast, increasing concentrations of Cr stress (30, 60, and 90 μg/g) led to a marked decline in IAA content. The lowest level (2.13 mg/g) was recorded under Cr-60 treatment, followed closely by Cr-30 (2.07 mg/g) and Cr-90 (2.29 mg/g), reflecting the inhibitory effect of heavy metal stress on endogenous phytohormone synthesis. Notably, the co-application of *C. tenuissimum* with Cr significantly restored IAA levels. The Cr-30 + *C. tenuissimum* and Cr-60 + *C. tenuissimum* treatments elevated IAA to 10.71 and 7.14 (3.72 and 3.60 mg/g), respectively. The Cr-90 + *C. tenuissimum* treatment resulted in 4.28 mg/g (27.38%).

Proline content, a key osmoprotectant, responded distinctively to Cr stress and fungal inoculation (Figure 4b). Among all treatments, the highest proline level (0.031 mg/g; 82.35%) was observed under Cr-90 stress alone, suggesting a strong stress response and activation of osmotic adjustment mechanisms. Similarly, in Cr-30 + *C. tenuissimum* and Cr-60 + *C. tenuissimum* treatments, proline levels were significantly reduced compared to Cr-only treatments.

**Figure 5:**
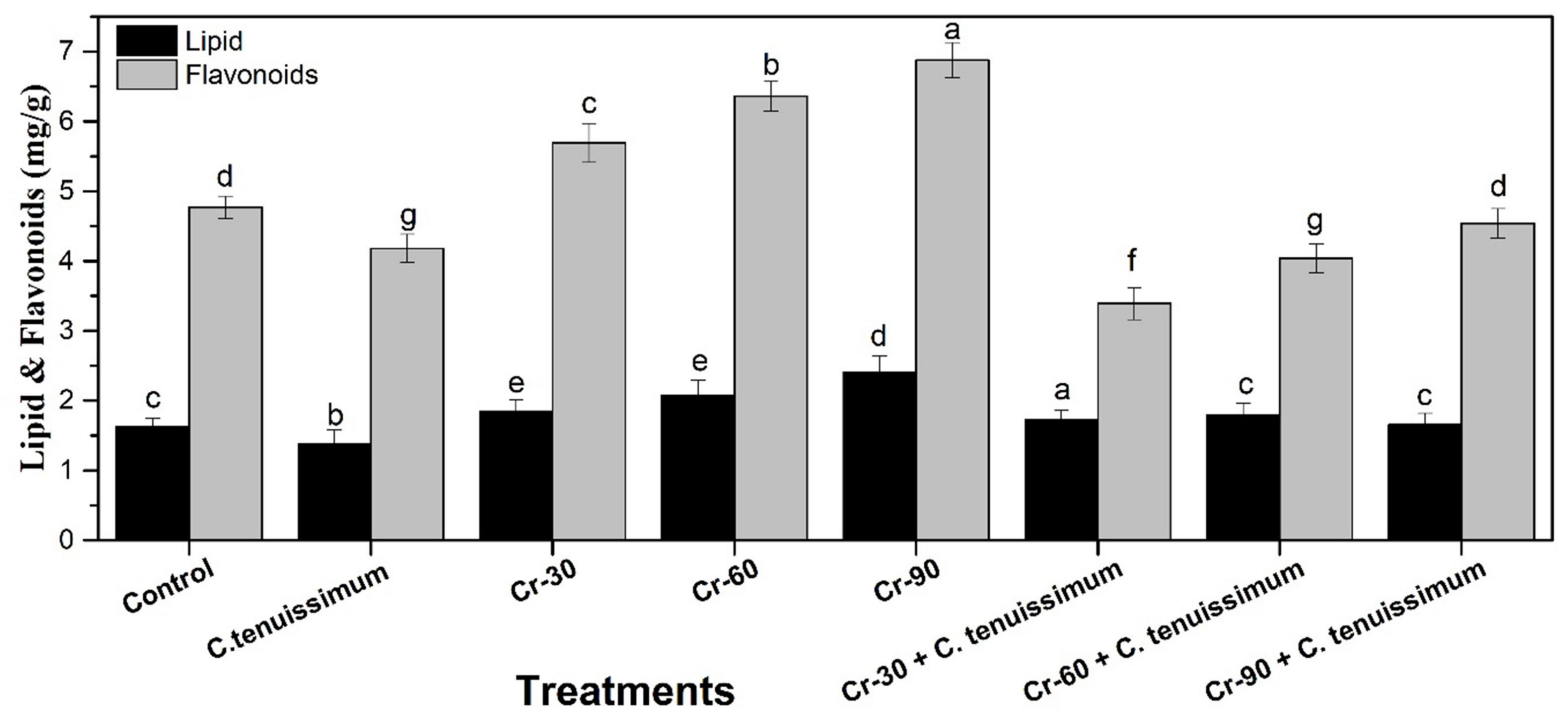
Effects of Cr with or without *C. tenuissimum* on flavonoids and lipid accumulation in wheat. Data are from three biological replicates, mean values ±SD.

The inoculation of *C. tenuissimum* resulted in a significant (*p* < 0.05) increase in lipid and flavonoid levels compared to the Cr-treated and control groups (Figure 5). Conversely, exposure to Cr stress alone resulted in concentration-dependent elevation of flavonoid levels. The highest flavonoid content (approximately 44.23% (6.8 mg/g)) was recorded under the Cr-90 treatment, indicating a notable increase relative to the control group. Lipid levels exhibited only slight variations across Cr-only treatments. Combined treatment with Cr and *C. tenuissimum* mitigated Cr-induced flavonoid accumulation. The Cr + *C. tenuissimum* groups (Cr-30 + CT, Cr-60 + CT, and Cr-90 + CT) exhibited lower flavonoid levels than their respective Cr-only treatments. Flavonoid content decreased from approximately 9.01% (4.34 mg/g) in treatment with Cr-90 + *C. tenuissimum*. Lipid levels in the combined treatments exhibited relative consistency or a slight increase compared to the control group.

### 3.3 Biological responses

#### 3.3.1 Effect of Cr and *C. tenuissimum* on peroxidase and superoxide dismutase activity in wheat

**Figure 6:**
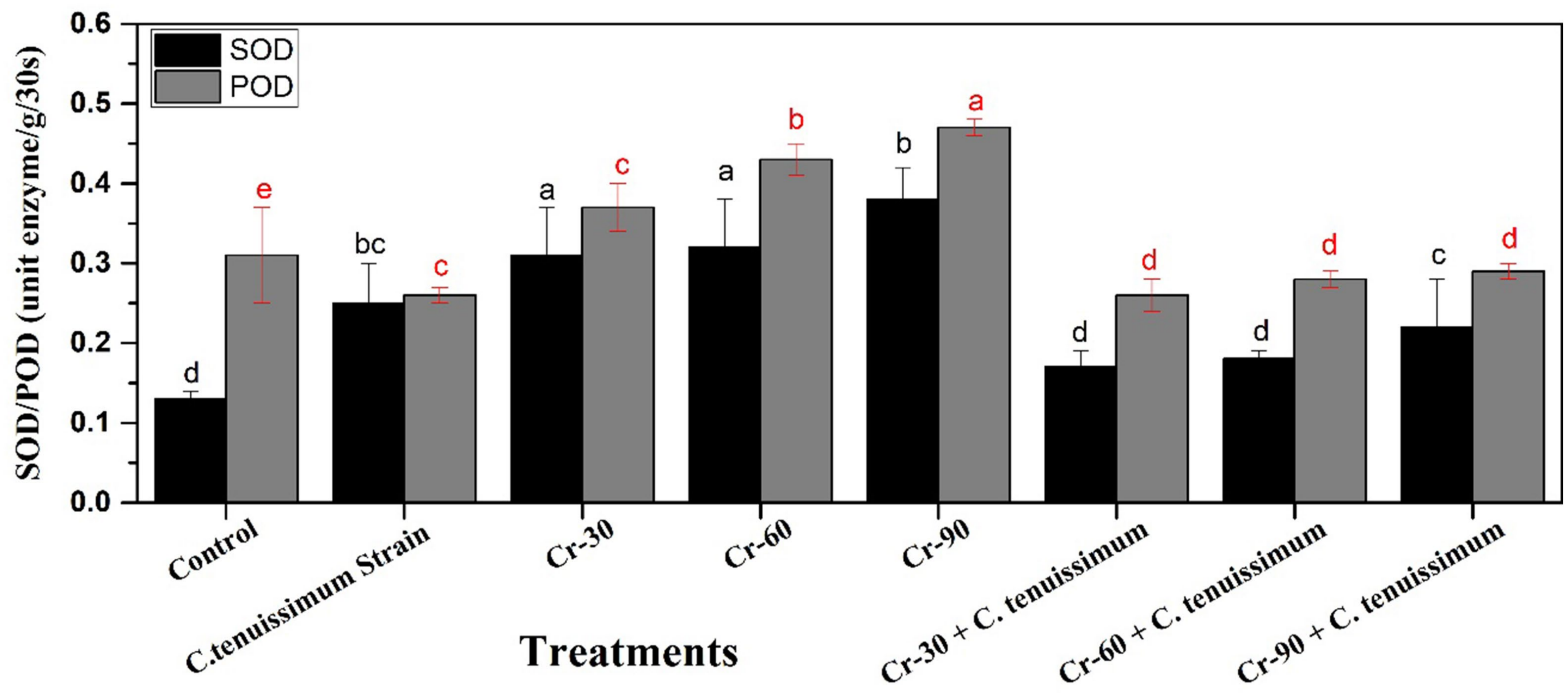
Effect of Cr and *C. tenuissimum* on superoxide dismutase (SOD) and peroxidase activities in wheat. Data are from three biological replicates, mean values ±SD.

Peroxidase (POD) and superoxide dismutase (SOD) activities were strongly affected by various treatments (Figure 6). All treatments resulted in a significant increase in SOD and POD activities compared to the control, with the most pronounced enhancements occurring under Cr stress alone. Among the Cr treatments, Cr + 90 demonstrated the highest activities of SOD at 0.39-unit enzyme/mg/3 s (26.30%) and POD at 0.53-unit enzyme/mg/3 s (51.61%), both of which were significantly greater (*p* < 0.05) than those observed in all other groups (Figure 6).

The application of the *C. tenuissimum* strain alone led to a modest but statistically significant enhancement in enzyme activities relative to the control group. The combined treatments with Cr and *C. tenuissimum* partially improved antioxidant enzyme activities. SOD and POD activities were significantly reduced in Cr + 30, Cr + 60, and Cr + 90 co-inoculated with *C. tenuissimum* compared to their respective Cr-only counterparts; however, these activities remained significantly elevated relative to the control group.

#### 3.3.2 Analysis of electrolyte leakage and catalase contents

**Figure 7:**
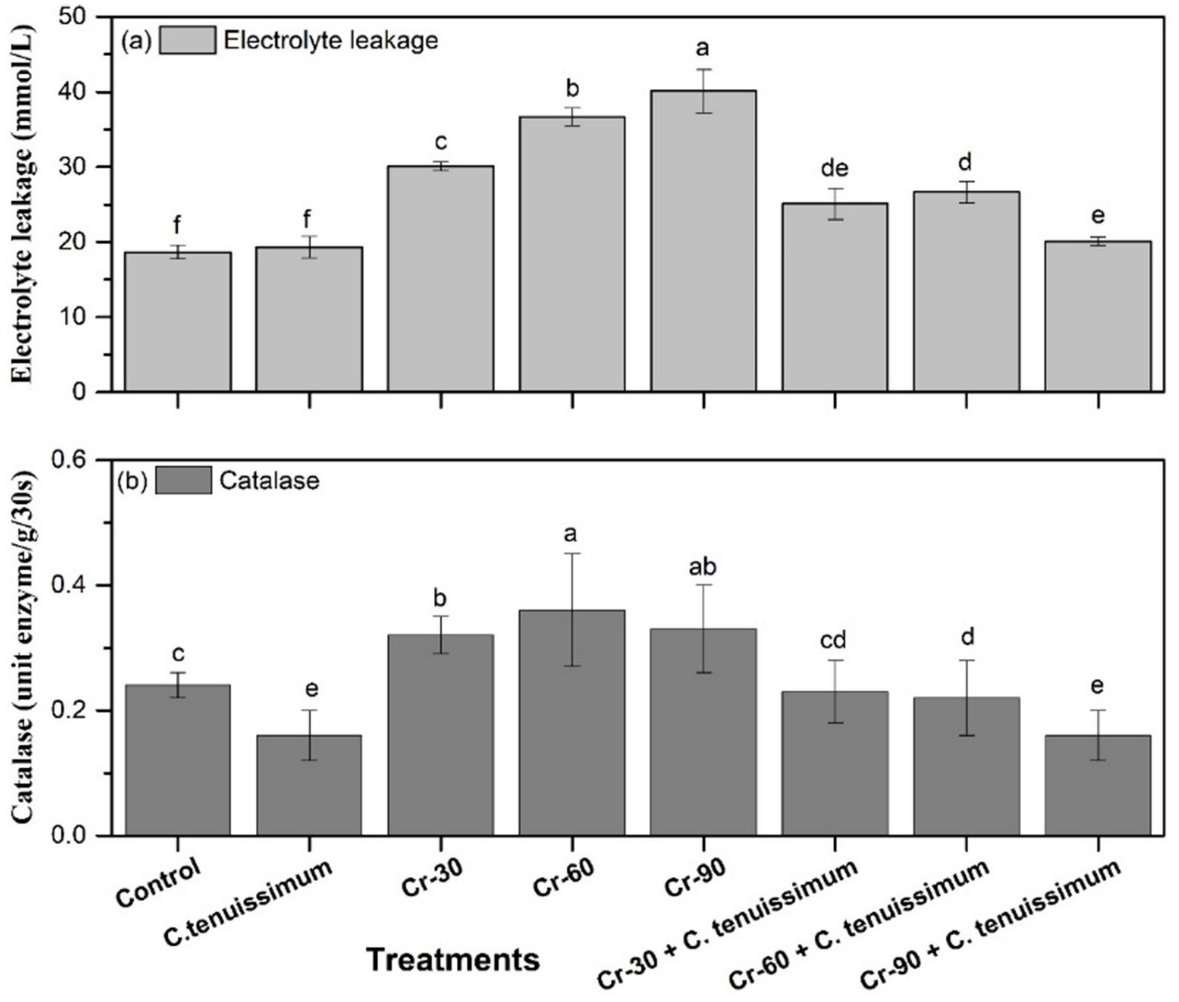
Concentrations of electrolyte leakage **(a)** and catalase **(b)** in wheat subjected to Cr stress with and without *C. tenuissimum*. Data are from three biological replicates, mean values ±SD.

In comparison to the control, electrolyte leakage (EL) increased significantly (*p* < 0.05) as a result of chromium (Cr) stress alone. Out of all the treatments, the Cr + 60 therapy had the greatest EL [around 62.84% (0.36 mmol/L), which was substantially higher (*p* < 0.05)]. Indicating membrane damage under Cr stress, Cr + 30 and Cr + 90 also caused significant increases in EL [69.48% (30.11 mmol/L) and 59.35% (39 mmol/L), respectively (Figure 7a)]. However, using *C. tenuissimum* either alone or in conjunction with Cr considerably decreased EL. There was no discernible difference between the control and *C. tenuissimum* treatments, which had the lowest EL values (both ~18.50 mmol/L). Although values were still higher than the control, the combined treatments (Cr + 30, Cr + 60, and Cr + 90 with *C. tenuissimum*) significantly decreased EL when compared to their respective Cr-only counterparts, indicating a protective function of *C. tenuissimum* in preserving membrane stability under Cr stress.

The catalase (CAT) activity pattern was similar to that of other antioxidant enzymes (Figure 7b). The Cr + 60 treatment exhibited the highest CAT activity at 0.38 U mg^−1^ FW s^−1^, followed by the Cr + 90 treatment at 0.33 U mg^−1^ FW s^−1^, and the Cr + 30 treatment at 0.32 U mg^−1^ FW s^−1^. The respective increases were approximately 44, 32, and 28% compared with the control group. Cr treatment alone significantly increased CAT activity. These values were substantially (*p* < 0.05) greater than those of the *C. tenuissimum* treatment (~0.15) and control (~0.22) (Figure 7). During Cr stress, co-inoculation with *C. tenuissimum* reduced CAT activity. Cr-60 and Cr + 90, in combination with *C. tenuissimum,* showed much lower CAT activity compared to Cr stress alone, but still greater than the *C. tenuissimum* treatment. According to these results, Cr stress causes oxidative stress reactions, as shown by the elevated EL and CAT activity. However, the application of *C. tenuissimum* counteracts these effects by improving membrane integrity and regulating the activity of antioxidant enzymes.

## 4 Discussion

The fungal strain exhibited consistent biomass production even at elevated Cr concentrations (Tatarin et al., 2024), indicating significant Cr tolerance during *in vitro* screening. Fungal species of the genera such as *Aspergillus*, *Penicillium*, and *Cladosporium* exhibit resistance to heavy metals through mechanisms such as metal binding, chelation, and sequestration (Singh et al., 2018; Girdhar et al., 2022; Abdel-Wareth, 2023). The isolate’s tolerance suggests its potential applications in the bioremediation of chromium-contaminated soils. The identification of the isolate as *C. tenuissimum* was confirmed through morphological and molecular characterization, with phylogenetic analysis indicating a close relationship with strains reported from China and India.

Cr stress alone significantly inhibited wheat growth, resulting in reduced root quantity and diminished root and shoot lengths. The observed negative consequences correspond with previous research indicating that Cr impedes root elongation, cell division, and the uptake of water and nutrients (Saud et al., 2022; Ullah et al., 2023). Despite Cr stress, inoculation with *C. tenuissimum* significantly improved all growth metrics, indicating a strong protective and growth-promoting effect (Răut et al., 2021). As previously indicated in other beneficial endophytic and rhizospheric fungi, this phenomenon may be attributed to the fungus’s ability to produce phytohormones, enhance nutrient solubilization, or modify stress-related gene expression (Devi et al., 2020; Cosoveanu et al., 2021; Adedayo and Babalola, 2023). *C. tenuissimum* in our study appears to alter several significant pathways at the molecular level in the presence of chromium. This process enhances the expression of antioxidant defense genes (SOD, POD, and CAT), promotes osmoprotective and detoxification pathways associated with proline and flavonoid metabolism, and stimulates the expression of auxin (IAA)-related genes that facilitate root growth and adaptation to stress. *C. tenuissimum* may influence the balance of metals in the plant body by activating proteins and transporters that bind to metals, potentially resulting in reduced chromium uptake and decreased toxicity. These combined chemical changes contribute to the maintenance of redox equilibrium, stabilization of membranes, and facilitation of normal metabolic and photosynthetic processes under stress conditions.

Wheat leaves exposed to Cr exhibited significantly reduced levels of carotenoids and chlorophyll, suggesting impairment of the photosynthetic apparatus. Heavy metals, such as Cr, disrupt chloroplast ultrastructure and pigment production (Balasaraswathi et al., 2017). This reduction was significantly diminished by *C. tenuissimum* inoculation, particularly in the Cr-60 + fungal treatment, suggesting its potential role in the maintenance of photosynthetic integrity. *Trichoderma, Penicillium,* and *Bacillus subtilis* species exhibit similar enhancements in pigment retention, nutrient uptake, and water use efficiency under drought stress when subjected to metal, drought, and heat stress following fungal inoculation (El-Mahdy et al., 2021; Syed et al., 2023; Zulkiffal et al., 2024; Ren et al., 2025). Under Cr stress, the levels of sugar and protein, key indicators of metabolic health, were significantly diminished, particularly at elevated doses. This aligns with evidence indicating that metal poisoning disrupts primary metabolism (Dubey et al., 2018; Wang et al., 2023; Asiminicesei et al., 2024).

The reduction in IAA levels observed under Cr stress aligns with previous research, indicating that HMs interfere with hormone signaling and biosynthesis. The notable increase in IAA levels following fungal inoculation suggests the ability of *C. tenuissimum* to synthesize auxin, a phenomenon also observed in other fungal endophytes and fungi that enhance plant growth (Suebrasri et al., 2020; Adedayo and Babalola, 2023). *C. tenuissimum* may alleviate stress by producing specific metabolites, including indole-3-acetic acid (IAA), proline, flavonoids, and antioxidant enzymes such as superoxide dismutase (SOD), peroxidase (POD), and catalase (CAT). These metabolites collaborate to facilitate cellular development, maintain homeostasis, and reduce oxidative stress in the presence of chromium. The observed increases in protein and IAA levels were likely attributable to both fungal biosynthesis and plant-induced production. *C. tenuissimum* produces IAA, which may facilitate root growth and activate plant hormone signaling pathways. Consequently, enhanced plant metabolism facilitated by fungi increases the production of IAA and proteins. This combination enhances plant development and improves resilience to stress induced by chromium exposure. The activation of osmoprotective responses, a prevalent plant defense mechanism in response to abiotic stress, was evidenced by the elevated proline levels observed in the Cr-alone treatments. Decreased proline levels in plants co-inoculated with the fungus suggest reduced oxidative and osmotic stress (Begum et al., 2022). Flavonoids play a role in reactive oxygen species (ROS) scavenging (Deng et al., 1997). Thus, Cr stress leads to increased flavonoid accumulation, indicating a heightened need for antioxidants. The reduction observed in the treated groups indicates that fungal protection diminished the oxidative burden. Consistent with research on other fungal-plant interactions, minor increases in lipid content during fungal inoculation may be associated with membrane stabilization and enhanced stress tolerance (El-Akshar et al., 2025; Chitra, 2002).

**Figure 8:**
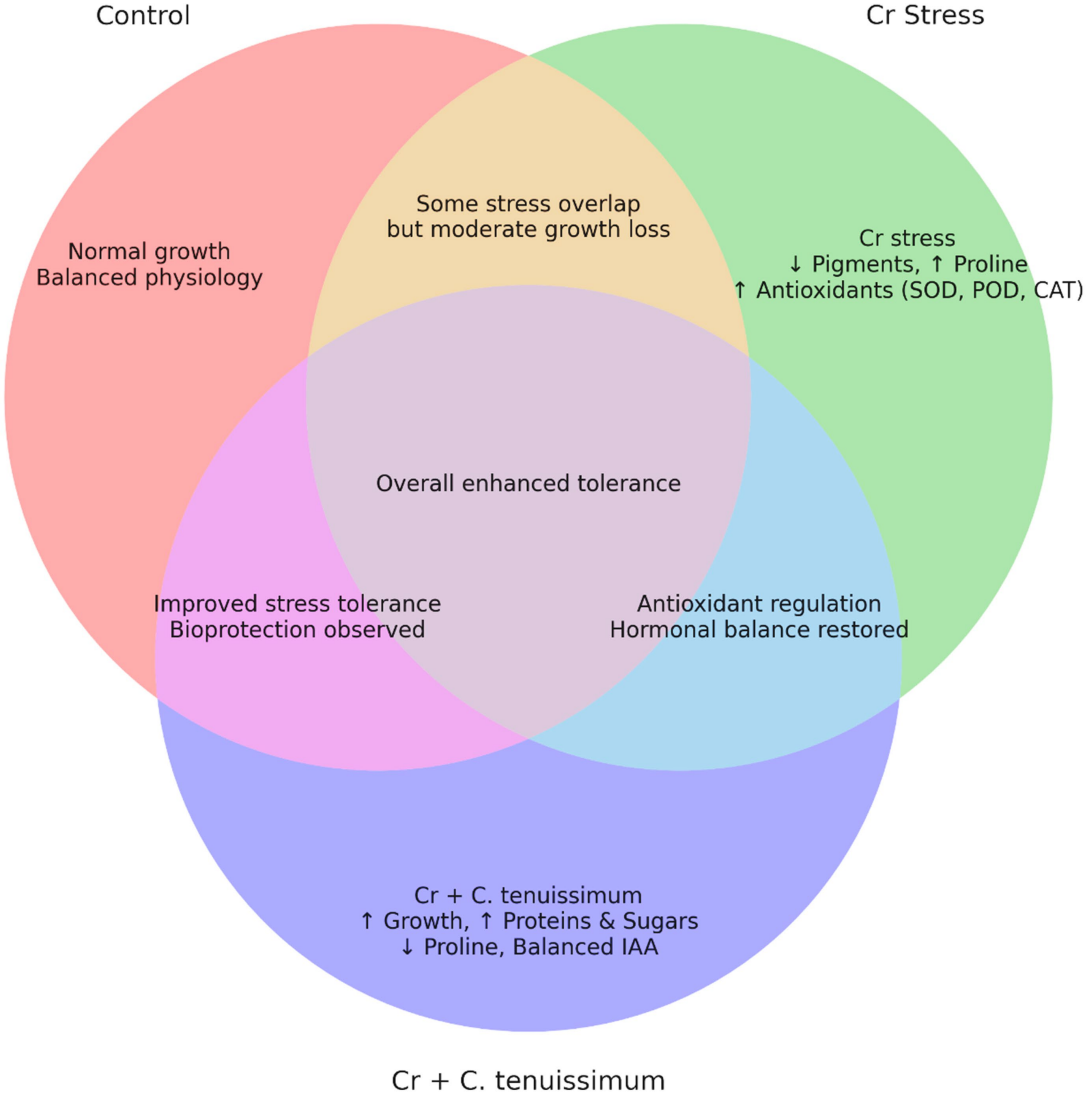
Venn diagram showing the effect of *C. tenuissimum* on *Triticum aestivum* under induced chromium stress.

The heightened activity of the key antioxidant enzymes SOD, POD, and CAT reinforces the hypothesis that Cr induces oxidative stress. Cr alone treatments exhibited the highest levels of enzymes, suggesting a stress response aimed at detoxifying reactive oxygen species (Kharbech et al., 2017; Malik et al., 2022). The inoculated groups exhibited moderate enzyme levels, indicating that the fungus partially mitigated oxidative stress and re-established cellular homeostasis, despite *C. tenuissimum* demonstrating minimal enzyme activity. Cr stress significantly elevated electrolyte leakage (EL), an indicator of membrane damage, whereas fungal-inoculated therapies substantially reduced EL. This suggests that *C. tenuissimum* plays a protective role in maintaining membrane integrity, potentially through enhanced antioxidant defense and reduced lipid peroxidation (Zhang and Feng, 2018; Zou et al., 2021). The observation that *C. tenuissimum* alters the antioxidant system to reduce the oxidative damage caused by Cr is further corroborated by the associated trend in CAT activity. Furthermore, the Venn diagram (Figure 8) demonstrates that several stress-responsive features were common to both the Cr-stressed and *C. tenuissimum*-treated groups. These findings revealed that *C. tenuissimum* performs a protective function by enhancing plant growth, pigment stability, metabolic processes, and antioxidant defenses to alleviate chromium toxicity.

## 5 Conclusion

This study highlights the importance of rhizosphere *C. tenuissimum* in fostering wheat growth and tolerance to Cr stress by boosting the levels of phytohormones, including proteins and IAA, reducing oxidative damage, and enhancing the activity of antioxidant enzymes. *C. tenuissimum* inoculation enhanced plant growth under stressful conditions by promoting longer roots and shoots, increasing biomass, and elevating chlorophyll concentration. The decrease in stress indicators, such as proline, lipids, flavonoids, and electrolyte leaks, provides additional evidence of the tolerance effect of *C. tenuissimum*. These findings indicate that *C. tenuissimum* has significant potential as a bioinoculant for sustainable agriculture, providing an effective approach to enhance crop yields and growth under challenging conditions. The integration of rhizospheric fungi into agricultural management practices represents a significant strategy for addressing the food security challenges posed by climate change. Further research is necessary to clarify the molecular mechanisms that govern plant-rhizosphere interactions, thereby enhancing their application in agriculture.

## Data Availability

The original contributions presented in the study are included in the article/supplementary material, further inquiries can be directed to the corresponding authors.

## Footnotes

1
http://www.ncbi.nlm.nih.gov/BLAST/
